# Adaptations to scale-up an early childhood education and care physical activity intervention for real-world availability — Play Active

**DOI:** 10.1186/s12966-023-01457-7

**Published:** 2023-06-01

**Authors:** Matthew Mclaughlin, Andrea Nathan, Ashleigh Thornton, Jasper Schipperijn, Stewart G. Trost, Hayley Christian

**Affiliations:** 1 Telethon Kids Institute, The University of Western Australia, Perth, Australia; 2 Division of Pediatrics, School of Medicine, The University of Western Australia, Perth, Australia; 3 Department of Sports Science and Clinical Biomechanics, University of Southern Denmark, Odense, Denmark; 4 School of Human Movement and Nutrition Sciences, The University of Queensland, Queensland, Australia; 5 School of Population and Global Health, The University of Western Australia, Perth, Australia

**Keywords:** Fidelity, Implementation, Childcare, Policy, Delivery mode

## Abstract

**Background:**

Adaptations for scale-up are ubiquitous but are rarely described in detail. Adaptations may be a key reason for the “scale-up penalty” which is when there is a reduction in intervention effect size following scale-up. The Play Active intervention consists of a physical activity policy for early childhood education and care (ECEC) services, with accompanying implementation support strategies. It was first implemented with 81 ECEC services in Perth, Western Australia, in 2021 — with significant positive changes in physical activity practice uptake. The aim of this paper is to describe the *extent, type, fidelity consistency, goals, size, scope, and proposed impact* of proposed adaptations to the implementation support strategies for scaling-up Play Active.

**Methods:**

Proposed adaptations were defined as planned changes, made prior to making the intervention available. The authors created a list of adaptations from a comparison of the Play Active implementation support strategies, before and after adaptation for proposed statewide availability across Western Australia, Queensland and South Australia, Australia. We used the Framework for Reporting Adaptations and Modifications-Enhanced Implementation Strategies (FRAME-IS) to code adaptations to implementation support strategies. Three authors coded each adaptation and rated their size, scope and proposed impact.

**Results:**

Fifty-three adaptations to Play Active were identified. Most (68%) were proposed for the ‘content’ of implementation strategies, including aspects of their delivery. In practice, this involved changing the delivery mode of implementation support strategies from phone call and email support, to website-based delivery. More than half (56%) of adaptations involved ‘adding elements’ for scale-up. Most adaptations were ‘fidelity consistent’ (95%). The main goals for adaptations were related to ‘increasing the acceptability, appropriateness, or feasibility’ (45%), ‘decreasing the costs’ (19%) and ‘increasing adoption of the evidence-based practice’ (19%). Adaptations were small to medium in size, with most proposed to have a positive (87%) or neutral (8%) effect on the effectiveness of the intervention, rather than negative (4%).

**Conclusions:**

A large number of small, fidelity-consistent, adaptations were proposed for Play Active scale-up. Overall, the process of reporting adaptations was found to be feasible. To understand the impact of these adaptations, it will be important to re-evaluate implementation, effectiveness and process outcomes, at-scale.

**Supplementary Information:**

The online version contains supplementary material available at 10.1186/s12966-023-01457-7.

## Introduction

Physical activity interventions must be delivered at-scale to achieve population-level health benefits [1, 2]. To address low-levels of physical activity [3, 4], a global call to action to scale-up physical activity interventions was made in 2018 by the World Health Organization (WHO) [5]. Scaling-up can involve moving research ‘along the pipeline’ from controlled research, to more real-world contexts, refining the intervention and demonstrating its implementation and impact in gradually larger and more ecologically valid contexts [6].

The scalability of public health interventions is defined as “the ability of a health intervention shown to be efficacious on a small scale and or under controlled conditions to be expanded under real-world conditions to reach a greater proportion of the eligible population, while retaining effectiveness” [1]. Systematic reviews have concluded that physical activity interventions have poor scalability with interventions initially trialed as randomized controlled trials typically losing 25–50% of their effect size when scaled-up [7–11]. Physical activity interventions for children may have even higher scale-up penalties, between 40 and 75% [11]. The reduction in effect size has been referred to as the “scale-up penalty” [12] and “voltage-drop” [13] which is of concern to the public health sector trying to roll out effective interventions at scale.

The reasons for scale-up penalty are poorly understood; however, inadequate intervention adaptations are thought to play a major role [14]. Interventions trialed as randomized controlled trials ubiquitously require considerable adaptations for scale-up [7–11], including interventions specifically focused on increasing physical activity [11]. Adaptations are planned [15] and can include changes to the evidence-based intervention itself, or to the implementation support strategies supporting the uptake of the intervention [15, 16]. Little is known about adaptations [15, 16], particularly adaptations to implementation support strategies made for scaling-up physical activity interventions [16].

Early childhood education and care (ECEC) is an ideal setting for physical activity promotion, with an umbrella review of systematic reviews suggesting that physical activity interventions delivered in ECEC generally improved children’s physical activity [17, 18]. In Australia, over 40% of children aged 0 to 5 attend ECEC and do so for an average 25 h per week [19]. Two in three West Australian children do not do enough physical activity while attending ECEC [4], with similar levels of inactivity reported in other Australian states and territory ECEC services [20].

Play Active is an ECEC-based physical activity policy intervention, with accompanying implementation support strategies [21–23]. Play Active was trialled and evaluated through a pragmatic cluster randomized trial (2021-22) [21–23]. Eighty-one participating ECEC services were randomly assigned to either the intervention or waitlisted comparison group. Data were collected from 565 ECEC educators pre- and post-intervention with a three-to-five-month policy implementation interval. Play Active resulted in significantly higher uptake of physical activity policy practices by educators (p = 0.034) [23]. Play Active had high awareness among educators (90%) and was acceptable to them (83%) [23]. Directors also reported high acceptability (78%) [23]. Fidelity and reach of implementation support strategies were also high (> 75%) [23].

In the ECEC sector, adaptations that address common barriers may improve intervention reach, implementation and effectiveness — such as staff-level barriers (e.g., high-workload, low-wages, time/money for professional development, and not feeling valued) [24] and ECEC service-level barriers (e.g., lack of workforce, lack of funding, difficulty engaging parents) [25]. Understanding adaptations is an essential component of evaluating scale-up research [26].

It is best practice in implementation science to describe the evidence-based intervention and the accompanying implementation support strategies separately [27]. The Framework for Reporting Adaptations and Modifications-Enhanced to Implementation Strategies (FRAME-IS) was recently developed to support the consistent documentation and reporting of adaptations to implementation support strategies [16], extending on the FRAME, which was developed to describe adaptations to the evidence-based intervention [15]. FRAME-IS provides a taxonomy of classifying adaptations to implementation support strategies, including *what* is adapted, the *nature* of the adaptation, *who* participated in the adaptation, for *whom*/*what* is the adaptation made and *when* it occurred. Despite the existence of FRAME-IS, adaptations to implementation support strategies are often poorly described [8, 10, 15, 28]. Furthermore, scale-up trials seldom report adaptations in detail, nor do they use consistent taxonomies, instead they rely on scant adaptation descriptions that fail to provide the details required to understand the reasons for potential scale-up penalties [15]. Only two settings-based interventions with children have to date used FRAME or FRAME-IS [29, 30], both in school settings. Research to understand what adaptations are needed in other child settings-based interventions (including ECEC) to improve young children’s physical activity levels at scale, is needed.

To address the lack of detailed adaptation reporting, a descriptive study of the adaptations proposed for the scale-up of an evidence based physical activity intervention (Play Active) targeting ECEC services was undertaken. The aim of this paper is to describe the *extent, type, fidelity consistency, goals, size, scope and proposed impact* of planned adaptations to the implementation support strategies of Play Active for scale-up, using an established taxonomy, FRAME-IS [16].

## Methods

### Ethical approval

Ethics approval was not required for this study. The Play Active pre-scale trial [21] was registered with the Australian New Zealand Clinical Trials Registry (reference number 12620001206910). The scale-up trial will be registered prior to commencement.

### Play active intervention

**Fig. 1:**
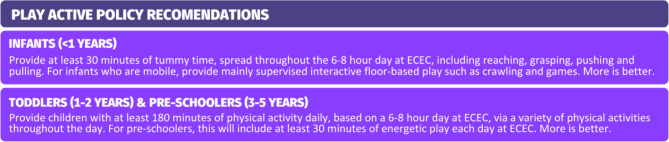
Play Active ECEC age-specific policy recommendations

The Play Active policy was developed in 2020 through a Delphi process [22] and evaluated through a pragmatic randomized trial conducted in 81 ECEC services in 2021-22 [21]. An outline of the evidence-based [22] physical activity policy recommendations for ECEC contained within the physical activity policy intervention is shown in Fig. 1. The policy, including 25 procedures to support the recommendations, will remain unchanged for scale-up, as the policy is, by design [22], evidence-informed, scalable and tailorable [31].

**Fig. 2:**
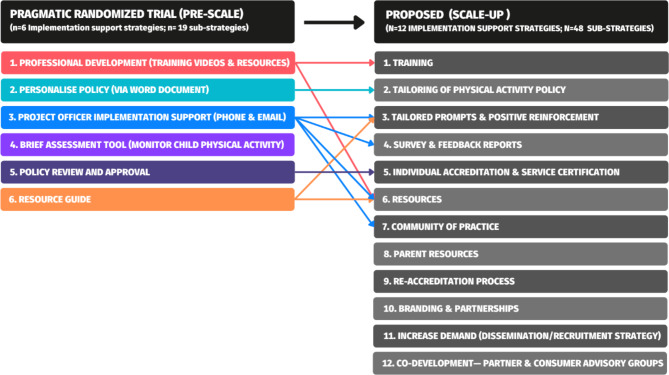
Overview of the adaptations to Play Active implementation support strategies from pre-scale pragmatic randomized trial, to those proposed for scale-up

The implementation support strategies offered to ECEC services during the pragmatic trial (pre-scale) are outlined in Fig. 2, alongside the ‘proposed’ intervention for delivery at scale (scale-up). The pragmatic trial consisted of six implementation support strategies (19 sub-strategies) delivered via email and phone calls with the research team [21]. The proposed scale-up of Play Active consists of 12 implementation support strategies (comprised of 48 sub-strategies). The major delivery mode proposed for scale-up is a custom-built website, to deliver the implementation support strategies.

#### Adaptation of implementation support strategies for scalable delivery

The purpose of scale-up adaptation was to design a highly scalable [31] version of Play Active that could be made available to all long daycare ECEC services statewide across Western Australia (n = 776), Queensland (n = 1,744) and South Australia (n = 445), Australia [32]. A detailed description of the scale-up adaptation process and a scalability assessment will be reported elsewhere. Briefly, adaptations were made in 2022 following the conclusion of the pragmatic trial [21]. The adaptation process was informed by the results of the four-step process of the ‘PRACTical planning for Implementation and Scale-up’ (PRACTIS) guide [33], as well as a scalability assessment [34] using the Intervention Scalability Assessment Tool [31]. Decisions about adaptations were guided by the Model for Adaptation Design and Impact (MADI) decision tree [28]. The process sought to retain the effects of the original intervention by maintaining fidelity consistency to the original implementation support strategies [21], while enabling greater reach (scaling up to ECEC services across three Australian states).

Briefly, The PRACTIS guide four-step process involved:

(i) Characterizing the parameters of the implementation setting – i.e., Western Australian, Queensland and South Australian long daycare ECEC services;
(ii) Continuing engagement with our Partner Advisory Group and Consumer Advisory Group (parents of young children) to inform decision making about adaptations;
(iii) Identifying contextual barriers and facilitators from our advisory groups as well as the pre-scale trial using qualitative and quantitative data from the pragmatic randomized trial in 2021 [23, 25]; and.
(iv) Addressing potential barriers to effective implementation. Throughout the adaptation process we prioritized scalability from a service-delivery perspective, considering workforce needs, affordability, practicability and acceptability [35].

Finally, the Play Active Partner Advisory Group (ECEC sector partners and educators, ECEC professional associations, Government partners, non-government health promotion partners, experienced researchers and Play Active research staff) reviewed the scaled-up implementation strategies and made the final judgement regarding the design and components of the resulting proposed scaled-up Play Active implementation support strategies.

### Measures for coding adaptations

We used the FRAME-IS framework to code all the adaptations made for delivering Play Active implementation support strategies at scale [15]. The FRAME-IS framework [16] comprises four core modules and three optional modules. The four core modules are: (1) brief description of the practice, implementation strategy, and modification(s); (2) what is modified; (3) what is the rationale for the modification; and for content modifications; and (4) what is the nature of the content modification and if the modification maintained fidelity to the core elements. The three optional modules in the framework are: (1) when is the modification initiated and is it planned; (2) who participates in the decision to modify; and (3) how widespread is the modification. Additional coding categories for proposed impact, size and scope were developed by the authors in line with suggested methods of Mark [36] and Rabin et al. [37]. Codes relating to the nature and timing of adaptations were, by design, the same across all adaptations (i.e., scale-up adaptations made prior to the delivery of the scale-up intervention) — as such, these codes from the FRAME-IS were not coded for each adaptation. Table 1 outlines the full list of coding and scoring for this study.

**Table 1:** Adaptation coding and scoring categories for Play Active

Adaptation Categories (reference derived from)	Code
Description of the adaptation	Free text
Reason for the adaptation (16)	Free text
What is adapted? (16)	□ Content (of the strategy itself, or how aspects are delivered) □ Training □ Context – Format □ Context – Setting □ Context – Personnel □ Context – Population
What is the type of content or training adaptation? (16)	□ Tailoring/tweaking/refining □ Changes in packaging or materials □ Adding elements □ Removing/skipping elements □ Shortening/condensing (pacing/timing) □ Lengthening/ extending (pacing/timing) □ Substituting □ Reordering of implementation modules or segments □ Spreading (breaking up implementation content over multiple sessions) □ Integrating parts of the implementation strategy into another strategy (e.g., selecting elements) □ Integrating another strategy into the implementation strategy in primary use (e.g., adding an audit/feedback component to an implementation facilitation strategy that did not originally include audit/feedback) □ Repeating elements or modules of the implementation strategy □ Loosening structure □ Departing from the implementation strategy (“drift”) followed by a return to strategy within the implementation encounter □ Drift from the implementation strategy without returning (e.g., stopped providing consultation, stopped sending feedback reports)
What is the relationship to the core elements (fidelity)? (16)	□ Fidelity Consistent/Core elements or functions preserved □ Fidelity Inconsistent/Core elements or functions changed
What is the goal? (16)	□ Increase reach of the EBP (i.e., the number of patients receiving the EBP) □ Increase the clinical effectiveness of the EBP (i.e., the clinical outcomes of the patients or others receiving the EBP) □ Increase adoption of the EBP (i.e., the number of clinicians or teachers using the EBP) □ Increase the acceptability, appropriateness, or feasibility of the implementation effort (i.e., improve the fit between the implementation effort and the needs of those delivering the EBP) □ Decrease costs of the implementation effort □ Improve fidelity to the EBP (i.e., improve the extent to which the EBP is delivered as intended) □ Improve sustainability of the EBP (i.e., increase the chances that the EBP remains in practice after the implementation effort ends) □ Increase health equity or decrease disparities in EBP delivery
At what level is the adaptation made? (16)	□ Organizational level (i.e., available staffing or materials)
When is the adaptation initiated (16)	□ Scale-up (i.e., when the EBP is being spread to additional clinics/settings within your system)
Is modification planned? (16)	□ Planned/Proactive (proactive adaptation)
Who participates in the decision to modify? (16)	□ Program Manager □ Implementer or implementation strategy expert (*Researcher)* □ Practitioners (*Childcare service providers, directors and educators)* □ Community members *(Parents)*
Indicate who makes the ultimate decision (16)	□ Program Manager
How widespread is the modification? (16)	□ Organization
Proposed impact of the adaptation on intervention effectiveness (43)	9-point scale from most negative possible (0) to neutral (5) to most positive possible (9).
Size of the adaptation (42)	9-point scale from smallest change possible (0), to small (3) to medium (5) to large (7) to largest change possible (9).
Scope of the adaptation across the entire implementation support strategy (42)	9-point scale from smallest change possible (0), to small (3) to medium (5) to large (7) to largest change possible (9).

Some FRAME-IS codes were amended to reflect Play Active terminology [16]

Abbreviations: EBP = Evidence-based practice

### Procedures

Three authors participated in the coding of adaptations closely following the instructions for using the FRAME-IS framework [16]. Firstly, MM identified a list of potential adaptations by comparing the list of pragmatic trial (pre-scale) implementation support strategies (see Additional File 1, Table 1) with a list of the proposed implementation strategies adapted for scalable delivery (see Additional File 1, Table 2) [21]. Forwards and backwards comparison between both lists of implementation support strategies generated 72 potential adaptations.

Three authors (MM, AN and AT) knowledgeable and involved in both the pragmatic trial [21] and scale-up process scored the adaptations. These authors met to initially familiarize themselves with each of the potential adaptations (n = 72) and the FRAME-IS codes (June 2022). MM first scored the adaptations independently. AN and AT then independently reviewed the potential adaptation codes identified by MM and either agreed or disagreed with the selected codes (June-July 2022). There were few disagreements in coding between the reviewers. To discuss disagreements and reach consensus, a short consensus meeting was held (July 2022). Following discussion, the coding for each of the adaptations was finalised.

The size, scope and proposed impact on the effectiveness of the intervention on a scale of zero through to nine were scored independently, in line with methods proposed by Mark [36]. Size and scope were scored from smallest change possible (zero) through to largest change possible (nine). Proposed impact of the intervention was scored from most negative possible (zero) through to most positive possible (nine).

### Analysis

Descriptive statistics for each adaptation category were calculated (Table 1). A final adaptation mean score was derived from the size, scope and proposed impact scores.

## Results

The results are reported by codes for adaptations, to address the *extent, type, fidelity consistency, goals, size, scope and proposed impact* of adaptations for Play Active scale-up.

### Extent (number of adaptations)

A total of 53 adaptations were coded across the 72 potential adaptations. Of the 72 potential adaptations, five adaptations were coded as not adapted (i.e., no changes were proposed for scale-up), and 14 adaptations were coded as previously coded (i.e., coded within another adaptation).

### Types of adaptations

#### What was modified?

Adaptations were made to content (n = 36, 67.9%), context (specific to format) (n = 8, 15.1%), training (n = 5, 9.4%) and context (other than format, setting, personnel, population) (n = 4, 7.5%).

#### What was the nature of the content or training adaptation?

More than half of adaptations involved adding elements (56.1%), for example, the introduction of a community of practice Facebook Group for educators and directors to share resources and ideas. Table 2 outlines the codes for the nature of the content or training adaptation.

**Table 2:** What is the nature of the content or training adaptation? Coding from the FRAME-IS

What is the nature of the content or training adaptation?^^^	n*	%
**Adding elements**	**23**	**56.1**
**Shortening/ condensing**	**5**	**12.2**
**Tailoring/ tweaking/ refining**	**5**	**12.2**
**Lengthening/ extending**	**4**	**9.8**
**Removing/ skipping elements**	**2**	**4.9**
**Substituting**	**1**	**2.4**
**Integrating another strategy into the implementation strategy in primary use**	**1**	**2.4**
**Total Adaptations**	**41**	**100.0**

*Does not sum to 53, as excludes not-applicable codes from the FRAME-IS (i.e., context adaptations)

^Several codes from module 3 of the FRAME-IS were not used in coding and are removed from this table (e.g., loosening structure)

### Fidelity consistency of adaptations

The vast majority of adaptations were fidelity consistent (95.1%). Two adaptations were fidelity inconsistent (4.9%). These fidelity inconsistent adaptations related to the addition of a fee for Play Active, which is an implementation support strategy designed to address accountability (buy-in) of ECEC services through payment [38].

### Goals of adaptations

The main goals for adaptations included increasing the acceptability, appropriateness, or feasibility of the implementation effort (45.3%); decreasing costs of the implementation effort (18.9%); and, increasing the adoption of the intervention (i.e., the policy) (18.9%). The goals of adaptations are outlined in Table 3.

**Table 3:** Goals of adaptations, coding from the FRAME-IS

What is the main goal of adaptation?^^^	n	%
**Increase the acceptability, appropriateness, or feasibility of the implementation effort**	**24**	**45.3**
**Decrease costs of the implementation effort**	**10**	**18.9**
**Increase adoption of the intervention**	**10**	**18.9**
**Improve sustainability of the intervention**	**5**	**9.4**
**Increase reach of the intervention**	**2**	**3.8**
**Improve fidelity to the intervention**	**1**	**1.9**
**Increase the clinical effectiveness of the intervention**	**1**	**1.9**
**Total Adaptations**	**53**	**100.0**

^Several codes from module 4 of the FRAME-IS were not used in coding and are removed from this table

### Size and scope of adaptations

The mean size of all adaptations was 4.2 (SD 1.6) out of nine, indicating that adaptations were generally small to medium changes. In total, there were 17 adaptations scoring greater than five out of nine, with only two adaptations scoring greater than seven. The largest adaptations (i.e., > 7) related to the introduction of the proposed member fee ($100 for 2-year membership). Five adaptations scored greater than six. These related to the policy audit being automated rather than led by a Project Officer, the ordering of implementation support such that training is compulsory to progress to policy tailoring, and the use of in-house training within the scale-up website rather than external training providers.

Similar findings were observed for the scope of adaptations. The scope mean was 4.3 (SD 1.5) out of nine, indicating that adaptations were generally small to medium in scope. In total, there were 17 adaptations scoring greater than five for scope, with only two adaptations scoring greater than 7. The largest scope adaptations (i.e., > 7) related to the proposed introduction of a member fee, aligning with the size and fidelity consistency findings.

### Proposed impact of adaptations

Of all adaptations, 46 were proposed to have at least somewhat of a positive impact (86.8%). Four adaptations were proposed to have a neutral impact and three a negative impact. Adaptations with a proposed positive impact included:

- providing online access to resources via a dedicated Play Active website, rather than printed materials alone;
- providing instant online and portable document format feedback to directors on their self-assessment of current practices at their service, rather than tailored feedback via email with a Project Officer;
- the creation of a community of practice Facebook group;
- providing short in-house training within the dedicated Play Active website, rather than external providers;
- making training mandatory as part of the policy tailoring;
- making it mandatory for directors to select at least 10 of 25 procedures in the policy tailoring to focus on over a two-year period, rather than three to five procedures;
- changing the policy tailoring process from a word document and email process, to a website-delivered guided form

Adaptations with a proposed negative impact, included the removal of free access to educator training and replacing the weekly project staff prompting phone calls to automated-tailored email and SMS reminders. Adaptations with a proposed neutral impact largely involved tailoring and tweaking, for example, housing resources within a dedicated Play Active website rather than elsewhere online.

## Discussion

### Principal Findings

This study is the first to code adaptations proposed to implementation support strategies for scale-up, using a consistent taxonomy – FRAME-IS [16]. This is important to advance the field of adaptation and scalability, where descriptions of adaptations for scale-up have been poor, which has posed a challenge with explaining the potential impact of adaptations on the scale-up penalty [11]. To address the lack of detailed adaptation coding, we coded 53 adaptations to Play Active’s implementation support strategies. Most of these adaptations (68%) were made to the way aspects of the implementation strategy content will be delivered. For example, changing the delivery mode of implementation support strategies from phone call and email support, to website-based delivery. More than half (56%) of adaptations involved ‘adding elements’ for scale-up, and only 17% involved removing or shortening elements. Most adaptations were ‘fidelity consistent’ (95%). The main goals for adaptations were related to ‘Increasing the acceptability, appropriateness, or feasibility’ (45%), ‘decreasing the costs’ (19%) and ‘increasing adoption of the evidence-based practice’ (19%). Adaptations were primarily small to medium in size and proposed to have a positive (n = 87%) or neutral (8%) effect on the effectiveness of the intervention, rather than negative (4%).

The adaptations coded as ‘fidelity inconsistent’, as well as large in size and scope, were related to the introduction of a membership fee for Play Active. Small intervention fees are costs that are not considered a barrier to implementation of physical activity interventions in ECEC [39]. The pragmatic trial (pre-scale) provided all implementation support strategies for free, but the proposed adaptation for scale-up introduces a AUD$100 fee (approx. USD$67) for two years, per service. The fee was introduced following discussion with our Partner Advisory Group during the scale-up process. The two main reasons to introduce the fee were to increase the sustainability of Play Active (i.e., by partly addressing concerns about funding) and to foster accountability (i.e., ‘buy-in’). Notably, Generation SunSmart® - a sun safety intervention delivered by our partner Cancer Council WA has achieved 95% coverage across Australian ECEC services using a similar per-service fee model [40]. Although these funds will not cover the full cost of delivering Play Active (e.g., project staff, website maintenance), the funds can cover the printed and physical materials that form a part of the membership pack. Given that lack of funding is a common barrier to sustainability of public health interventions [41], the introduction of a small fee may be an appropriate strategy for sustainability and feedback from our ECEC partners has confirmed that this membership fee is considered very feasible, relative to other biennial memberships costs. Future efficacy research may consider introducing a small fee for the implementation support strategy, to reduce the number of fidelity-inconsistent adaptations required for scale-up [28, 33].

Similar to a previous study exploring the extent, type and reasons for adaptations to a secondary school physical activity intervention using the FRAME [15, 30], our adaptations were largely coded as fidelity consistent, and most were proposed to have a positive effect. Both this study and the current study [30] involved the original trial developers, as well as partners, in the adaptation process. Both studies also retained the original delivery organization as the scale-up delivery organization [31]. Using the same delivery organization may also help to reduce the number of fidelity inconsistent adaptations. As such, future interventions should consider trialing interventions within the delivery organizations that have the capacity to deliver the intervention at-scale.

Prior research optimizing an implementation strategy supporting the uptake of a physical activity policy in schools used the FRAME-IS; however, this study only documented one overall adaptation to the implementation support strategy [29]. Our work extends this work by coding each individual adaptation (n = 53) separately, to highlight the *extent, type, fidelity consistency, goals, size, scope and proposed impact* of each adaptation proposed for scale-up. Another study coded implementation and intervention adaptations to a weight management program [42]. They found that their fidelity consistent adaptations (e.g. face-to-face delivery of implementation support to online delivery) were able to retain effectiveness when re-evaluated [42]. Given our high-level of fidelity consistency (95%), we will be able to evaluate the oft-cited recommendation included within adaptation and scale-up guidance [8, 43, 44] – that fidelity consistent adaptations may be more likely to retain effectiveness than non-fidelity consistent adaptations. It will be important to re-evaluate the effectiveness and implementation of Play Active at-scale.

The next logical step for Play Active is to consider how adaptations will impact intervention and implementation outcomes using the Model for Adaptation Design and Impact (MADI) [28]. This would involve conducting a scale-up trial using these proposed implementation support strategies. It’s unclear if this approach of coding each individual adaptation is more suitable for understanding the future impact of adaptations, or if it may be better to package similar adaptations together [45]. For example, examining adaptations in clusters may provide greater insight about how adaptations function in a system, including how they interact to influence outcomes [45]. Additionally, to our knowledge, this is the first paper to code size and scope of intervention adaptations [36]. This size and scope data may be particularly useful to understand the impact of adaptations. It is has been hypothesized that larger adaptations have the potential for greater impacts than smaller adaptations [36]. Our paper provides an opportunity to explore how size and/or scope of adaptations influences the impact of adaptations and modifications empirically, in the future.

### Feasibility of using FRAME-IS

Describing the adaptations using the FRAME-IS framework was deemed feasible and acceptable by the research team and is considered best practice [16]. The research team from the pragmatic trial was involved in the adaptation coding process. This meant that the team were very familiar with the pragmatic trial, so could easily code the adaptations proposed for scale-up. Secondly, detailing the implementation support strategies in considerable detail (Additional file 1) enabled clear identification of the differences between the pragmatic trial (Additional file 1, Table 1) and the proposed scaled-up version of Play Active (Additional file 1, Table 2).

### Strengths, limitations, implications for future research

The main strength of this paper is the prospective reporting of proposed implementation support strategy adaptations, in detail, to outline their *extent, type, fidelity consistency, goals, size, scope and proposed impact*. To our knowledge, no other study has used the FRAME-IS to code adaptations proposed for scale-up, following a comprehensive adaptation process with stakeholders. Another strength is the addition of size, scope and proposed impact ratings to the FRAME-IS coding [36]. Such ratings allowed adaptations that were ‘larger’ than others, but importantly highlighted that most adaptations were small to medium in size [36].

Despite these strengths, there were two key limitations. Firstly, further adaptations to the implementation support strategy may be required for rural, remote, culturally and linguistically diverse ECEC services, and services with higher proportions of Indigenous children. Such adaptations can occur during the proposed delivery of the scaled-up implementation support strategy, based on feedback from ECEC services. It is therefore important to prospectively track modifications and adaptations during scale-up intervention delivery. Secondly, adaptations to the evaluation plan were not described. Once the evaluation methods and design of a scale up trial have been fully confirmed, it will be important to apply the FRAME to code adaptations to the evaluation design.

## Conclusions

This study describes the *extent, type, fidelity consistency, goals, size, scope and proposed impact* of Play Active adaptations. Our findings highlight that a large number of small and fidelity consistent adaptations are proposed for scale-up of Play Active. The vast majority of adaptations were coded to have a proposed positive impact for scale-up. Such data are important to aid in the interpretation of Play Active and other scale-up trials findings. Finally, to understand the impact of these proposed adaptations, it will be important to conduct a scale-up trial.

## Electronic supplementary material

Below is the link to the electronic supplementary material.

Additional File 1: Table 1 Play Active implementation support strategies from the efficacy trial and those proposed for a scale-up trial. Table 2 Play Active implementation support strategies proposed for scale-up trial statewide across Western Australia, Queensland and South Australia

## Data Availability

Not applicable.

## Abbreviations

ECEC: Early Childhood Education and Care
FRAME: Framework for Reporting Adaptations and Modifications-Enhanced
FRAME-IS: Framework for Reporting Adaptations and Modifications-Enhanced - Implementation Strategies
MADI: Model for Adaptation Design and Impact
PRACTIS: PRACTical planning for Implementation and Scale-up
