# Supplementary material for: Adaptations to scale-up an early childhood education and care physical activity intervention for real-world availability — Play Active
Source: Int J Behav Nutr Phys Act. 2023 Jun 1;20:65. doi: 10.1186/s12966-023-01457-7 (PMC10233970; doi:10.1186/s12966-023-01457-7)
Supplement: Supplementary file 1 — Additional File 1: Table 1 Play Active implementation support strategies from the efficacy trial and those proposed for a scale-up trial. Table 2 Play Active implementation support strategies proposed for scale-up trial statewide across Western Australia, Queensland and South Australia [file 12966_2023_1457_MOESM1_ESM.docx]

# **Additional File 1**

**Table 1:** Play Active implementation support strategies from the efficacy trial (1)

| **Implementation support strategy** | **Itemized components (sub-strategies)** |
| --- | --- |
| 1. **Personalise policy** | 1. ECEC services are encouraged to **tailor** the physical activity policy template to their service and asked to do this within the first 2 months of receiving the policy template. This serves to enhance **policy** **ownership**. This also aligns with the program being an **adaptable** intervention |
|  | 1. To assist ECEC services to tailor their physical activity policy they are asked to include at least **five** practices (from the 25 in the policy template) into their own physical activity policy^1^ |
|  | 1. **Seven** **prioritized** high-impact and low-effort practices are recommended to services. They are: 2. ensuring awareness and understanding of the physical activity policy; 3. providing many daily opportunities for outdoor playtime; 4. providing opportunities for children to engage in discovery learning and discussing the importance of physical activity 5. breaking up prolonged periods of sedentary behaviour (e.g., sitting or standing for long periods or confining infants to highchairs or cots when not eating or sleeping); 6. not using punitive measures such as withholding physical activity as punishment for managing challenging behaviours (e.g., seated time out) and not using physical activity as punishment (e.g., star jumps); 7. wearing comfortable and appropriate clothing and footwear that doesn’t limit children’s and educator’s ability to engage in physical activity; 8. making the physical activity policy available to staff, families and visitors. |
| 1. **Policy review and approval** | 1. Policy **audit** and **feedback** – after submission of the draft policy by the service, the policy is reviewed by two Project Officers and tailored feedback is provided within one week of submission if the draft policy doesn’t meet the required standards^2^ |
|  | 1. If the policy meets the required standards^2^, **approval** is given for the policy and the remaining implementation support strategies are provided |
|  | 1. After approval**,** a **poster** is sent to each service that includes their selected practices (from five to 25 practices, depending on what they selected (see 1.2)) |
| 1. **Resource guide** | - 1. An 89-page resource guide is provided as a **hard copy (bound) and digital PDF** version to services |
|  | - 1. A QR code within the document links to a ‘**link tree’** of digital resources, including: a 2-minute Play Active video, a 20-minute Play Active educator webinar, expression of interest link for Play Active and links to all of the digital resources mentioned in the resource guide (48 total) |
|  | - 1. The resource guide is **mapped** to the 25 practices^1^ outlined in the policy, expanding on the content in the policy template, and acting as a specific reference for each of the 25 practices |
|  | - 1. The guide is **consistently structured** into three sections for each procedure: (i.) What can you do; (ii.) What does it mean; (iii.) Helpful resources |
|  | - 1. **Credibility** features are included in the resource guide (e.g., government and non-government partner endorsement, Minister for Community Services foreword) |
|  | - 1. Links made to ECEC National Quality Standards (where relevant) throughout the guide – **policy alignment** |
| 1. **Brief assessment tool** | - 1. **Practice 9** (of 25 practices in the physical activity policy) is the Energetic Play Assessment Tool. Within four standardised periods of the day (arrival to morning tea, to lunch, to afternoon tea, to departure) the degree to which each child engages in energetic play is scored (five-point scale: very rarely energetic to very often energetic) by educators. |
| 1. **Professional development** | - 1. **Practice 12** (of the 25 practices in the physical activity policy) outlines educators taking part in professional development to increase knowledge and skills around supporting children’s physical activity |
|  | - 1. KIDDO evidence-based online training: Six 30–60-minute modules including resources, training, quizzes and assessment. Content is focused on developing educators’ skills to develop students’ fundamental movement skills. Access to the training is free for trial services and made available to educators via email. |
|  | - 1. Nature Play WA evidence-based online training: Five modules, each approximately 3-4 hours long, to be completed at service level over 4-6 weeks. Content is focused on the importance of being a playful educator; active and playful outdoor learning environments; using outdoor play to increase physical activity; planning cycles for active outdoor play; and documenting as a process of reflection, intention and communication. Educators encouraged to complete this training through the resource guide and the Project Officer. The training has a cost of AUD$49 per module, total cost AUD$686. |
|  | - 1. Nature Play WA evidence-based face to face training: Services are offered a 2-hour face to face training course, at a cost of AUD$550. Content is an abridged version of the Nature Play WA online training. |
| 1. **Project Officer implementation support** | - 1. Weekly phone call and/or email to remind services to submit their draft policy for review and approval. |
|  | - 1. A mid-implementation (6 weeks) check-in phone call made by the Project Officer to the services to determine whether policy implementation has commenced. |

**Footnotes**

^1^The 25 practices are expanded to 27 practices in the policy template, as two additional practices are added which are also implementation support strategies (policy review and approval; the resource guide).

^2^ The minimum requirements for physical activity policies to be approved are that it includes: two (out of two) key statements; nine (out of nine) physical activity and sedentary behaviour recommendations; and at least five (out of 25) practices to prioritise implementing during the trial’s intervention period.

**Table 2:** Play Active implementation support strategies proposed for scale-up trial statewide across Western Australia, Queensland and South Australia (1)

| **Implementation Support Strategy (n=12)** | **Itemized components (implementation support sub-strategies n=48)** |
| --- | --- |
| 1. **Training** | 1. (Educators and directors) Play Active training videos, designed to be completed via mobile 2. Eight state-specific **free short videos** (60-90 seconds each) delivered online via Play Active website (Western Australia, Queensland and South Australia each has their own eight videos, to reflect their unique state-specific contexts. The content of the videos is consistent between states.). 3. Videos designed to **address specific barriers and facilitators:**  - Why is physical activity important? - Creating environments for energetic play? - Weather and active play - Nature play - How to become a Play Active centre - How to use the indoor environment for physical activity - Becoming a positive role model - Talking to families about physical activity  1. After each video, a single question to assess knowledge gained from the video 2. Once training videos are completed, the ECEC staff member receives a personalised certificate identifying them as a “Play Active Champion”. They are then encouraged to proceed to the next module in the website (tailored physical activity policy) |
|  | 1. Completion of all of the initial training videos modules unlocks access to access to the editable policy template |
| 1. **Tailoring of Physical Activity Policy** | 1. The director completes the policy on behalf of their service, the time expected to complete this step is 10 minutes. Through a series of prompted questions, one person from each service starts to "build your own policy". This online policy development process uses forced compliance technique to ensure the policy aligns with minimum standards outlined in Play Active policy. Services must select a minimum of 10 of the 25 practices to prioritise in their policy. By completing the policy, Directors agree to mandate change in their service in line with the policy. |
|  | 1. As part of the membership pack, services have access to download their completed physical activity policy as a PDF and are sent a printed copy within the mail-out pack. |
| 1. **Tailored Prompts & Positive Reinforcement** | Automated prompts (both email and SMS) are sent for either an action (e.g., positive reinforcement congratulations email for completing their policy) and an ‘in-action’ (e.g., to prompt to complete training). |
|  | 1. At all stages within the website, specific and tailored automated follow up emails and/or SMS messages are sent. These may be to prompt an action (email or SMS) or to provide resources (email). Automated messages will be sent at least weekly to registered users. |
|  | 1. At multiple stages within the website, short tailored automated follow up SMS messages are sent when email prompts fail to lead to desired outcome. |
| 1. **Survey & Feedback Reports** | 1. One ECEC staff member from each service, the first to register, will be prompted to complete an initial baseline survey prior to completing training. The survey contains 25-items about the services current uptake of Play Active practices and additional items to assess the effectiveness on their children’s moderate to vigorous-intensity physical activity. |
|  | 1. At 12 months and 24 months following registration, the same staff member will be prompted to complete the survey again. If there is no response from that staff member within 1 month and three follow-up prompts, then any other staff with a registered account in the website associated with the same service will be asked to complete the survey instead. |
|  | 1. Immediate feedback provided via email as a report, summarising their score and areas for improvement, generated based on the responses in the survey. |
| 1. **Individual Accreditation & Service Certification** | 1. Individuals (e.g., ECEC staff, including educators and Directors) receive a “Certificate of Individual Accreditation” for completing the Play Active training. |
|  | 1. The number of individuals completing training is added to the homepage of the website. |
|  | 1. Individuals completing training are searchable on the Play Active website via an online database (displaying name and service only). If individuals (educators or Directors) change services, their account details can be easily self-updated to reflect this. |
|  | 1. Services receive a “Certificate of **Accredited Play Active Service”** when at least one educator or director has completed the initial training, and the Director has completed the policy personalization and paid the membership fee of $100 for two years. |
|  | 1. The number of accredited services is added to the home page of the Play Active website. |
|  | 1. Accredited services are added to a map on the Play Active website |
|  | 1. Physical Membership Pack is posted to service 2. Resource Guide (bound) 3. ‘Play Active Certificate with Service name 4. Energetic play assessment tool (EPAT) 5. ECEC physical environment checklist 6. Parent engagement resources 7. Play Active information posters about play to display within service 8. Play Active sign/plaque for outdoor wall 9. Four round Play Active floor decals |
|  | 1. Service receives **digital access** via the Play Active website to all paper-based resources (housed on Resources section of members area of website, as well as being sent via email) |
|  | 1. State-specific **n**ewsletter sent out monthly to members (all registered emails linked to service) – including suggested activities and resources. National content for the newsletter is used for all three states, Western Australia, Queensland and South Australia. |
| 1. **Resources** | 1. Member services receive digital PDF copy of 89-page resource guide and hard copy (bound) |
|  | 1. For members, all resources referenced in the resource guide are housed on the Play Active website under a ‘Resources’ tab |
|  | 1. Resource guide is **mapped to the 25 practices** outlined in the policy template |
|  | 1. Resource guide also split into resources for each practice and added to the resources page on the Play Active website separately |
|  | 1. Resources aligned to the selected policies within the services policy are emailed monthly to members |
|  | 1. The guide is **consistently structured** into three sections for each procedure:   (i.) What can you do?; (ii.) What does it mean?; (iii.) Helpful resources.   1. Online resource guide tabs are consistently structured in the same way |
|  | 1. Links made to ECEC National Quality Standards (where relevant) throughout the guide – **policy alignment**. |
|  | 1. Resource guide content is delivered on state-specific Facebook Groups as weekly content posts (via content management system e.g., Buffer) |
|  | 1. **Practice 9** (of 25 practices) is the Energetic Play Assessment Tool (EPAT). Within each period of the day the degree (5-point Likert scale) to which each student is energetic is scored by educators. A 1-page guidance sheet is available via the resources tab for ECEC staff to interpret the EPAT scores |
|  | 1. ECEC Physical Environment checklist 2. **Practices 15-18** refer to the ECEC physical environment. The Physical Environment checklist includes 5 domains with 12 checkpoints for services to assess their physical environment. 3. The Physical Environment checklist includes links to achieving ECEC National Quality Standards |
| 1. **Community of Practice** | 1. Accredited services gain access to a state-specific **Accredited** **Play Active Members Facebook Group** for Western Australia, Queensland and South Australia, respectively. 2. The Champions Group encourages sharing of successes, challenges, ideas, and support among services 3. Discussion facilitated and encouraged by state co-located Play Active Project Officer |
|  | 1. Services are prompted monthly within the newsletter and **asked to share their positive experiences** of Play Active within the Accredited Play Active Members Facebook Group |
|  | 1. Educators and Directors are encouraged to ask questions within Facebook Group. They also have access to “Send Message” function within the Facebook Group which leads directly to a Play Active Project Officer, who spends one-two hours per weekday responding to messages (i.e., one day per week). |
|  | 1. Lapsed members are removed from the Facebook Groups after 3 months of lapsed membership and six follow-up prompts about re-accrediting as a member of Play Active. An auto-generated email is sent from the website to the Project Officer to remove the members. |
| 1. **Parent Resources** | 1. Parent resources section on website, includes a video that encourages parents to get active with their kids as well as general information about play. |
|  | 1. Training video 8 addresses how to engage with parents about Play Active and play, including energetic play specifically. |
|  | 1. Parent information poster provided to services to display in foyer, including links/QR codes for parents to follow to subscribe to Play Active Newsletter and follow Play Active Facebook page |
|  | 1. Play Active newsletter for parents – sent monthly by Play Active Project Officers. This newsletter is not state-specific, it includes activities from across the three states. |
|  | 1. A public Play Active Facebook Page – managed by Play Active Project Officer. This public Facebook Page is not state-specific, it includes activities from across the three states. |
| 1. **Re-accreditation Process** | 1. **Automated email** sent to services 12, 8, 4, and 2 weeks before membership expiration, and 1,2, 4, 8, 10 and 12 weeks after membership lapse. The email will be auto tailored to the service’s prior usage of member benefits, to highlight the benefits of membership to them. |
|  | 1. To **become re-accredited** services, pay reaccreditation membership fee ($50 for two years) |
|  | 1. Re-accredited services are prompted to immediately review their policy, update and expand on their prior policy. |
|  | 1. Eight additional Play Active specific professional development modules, including 5-minute training videos, links to resources, and a three-question quiz after each video |
|  | 1. Lapsed services removed from map of accredited services on website and membership access to resources section and additional training is revoked. |
| 1. **Branding & Partnerships** | 1. Partner logos, including evaluation partner logos, to be displayed on the website and where feasible on printed resources. |
|  | 1. Create brand guidelines and communications pack to guide decision-making about the use of the Play Active brand. |
|  | 1. Form written partnerships with partners that outline ways of operating and cross-promoting Play Active, including resource sharing agreements and statements on how partners will do to help implement and/or disseminate the intervention. |
| 1. **Increase Demand (Dissemination/ Recruitment Strategy)** | 1. The dissemination strategy is designed to reach new services not yet registered in the website, to create demand for registrations in the website (the dissemination goal). This will include, for example, paid social media advertising and media releases about the program. Such activities are expected to create a positive reinforcing reputation for the Play Active brand. The dissemination strategy may interchangeably be referred to as “recruitment strategy” for research purposes. |
| 1. **Co-development – Partner & Consumer Advisory groups** | 1. Community Stakeholder Group meets quarterly, a group of parents and community members are convened by Play Active to discuss the program and potential modifications that can be made to improve it. |
|  | 1. Partner Advisory Group meets quarterly, the group of partners and supporters meets to provide input and advice on implementation efforts and governance to any suggested modifications. |
